# Fast crystallographic texture mapping of atomically thin hBN films on Ni(111) using secondary electron contrast†

**DOI:** 10.1039/d5na00457h

**Published:** 2025-07-02

**Authors:** Vitaliy Babenko, Se Hun Joo, Anastasia P. Krief, Evan Tillotson, Sarah J. Haigh, Chris J. Pickard, Stephan Hofmann

**Affiliations:** a Department of Engineering, University of Cambridge Cambridge CB3 0FA UK sh315@cam.ac.uk; b Department of Materials Science & Metallurgy, University of Cambridge Cambridge CB3 0FS UK; c Department of Chemical and Biological Engineering, Sookmyung Women's University Seoul 04310 Republic of Korea; d Department of Materials, University of Manchester Manchester M13 9PL UK; e Advanced Institute for Materials Research, Tohoku University Sendai 980-8577 Japan; f Center for Science and Innovation in Spintronics, Tohoku University Sendai 980-8577 Japan

## Abstract

High-throughput characterisation and fast sample quality feedback loops are essential for accelerating the development of advanced materials and their technological applications. This is particularly pertinent to the chemical vapor deposition (CVD) driven industrialisation of ultra-thin device materials, like hBN, where a complex texture at the atomic layer level remains hard to characterise efficiently, particularly over large areas. Here, we show that secondary electron contrast can be used to fingerprint the alignment of monolayer hBN domains with the Ni(111) surface, thus opening fast crystallographic texture mapping capabilities with conventional scanning electron microscopy (SEM). Density functional theory (DFT) computations indicate a work function difference of 50 meV between the two possible epitaxial hBN orientations and around 600 meV between epitaxial and non-epitaxial (rotated) hBN orientations, where we also account for vicinal Ni surfaces. Through self-consistent secondary electron contrast assignment, we demonstrate the effective and systematic analysis of the large-area structure of hBN CVD films and how this can provide insight into their growth process and evolution, including the nature of the resulting domain boundaries, adlayers and overlapping bilayer regions.

## Abstract 2

Fast crystallographic texture mapping of monolayer films with conventional scanning electron microscopy, including the nature of the resulting domain boundaries, adlayers and overlapping bilayer regions.

## Graphical abstract

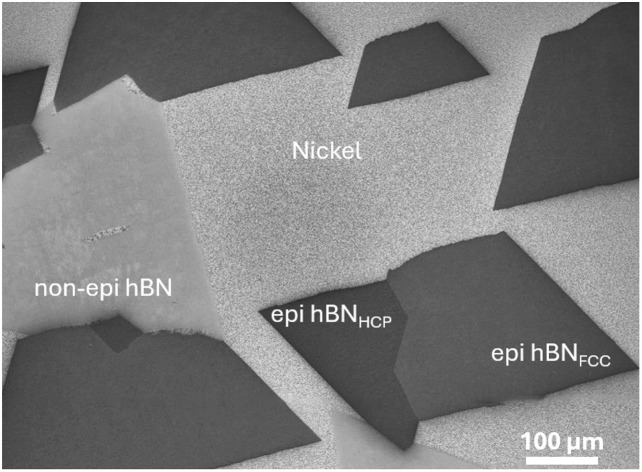

## Introduction

Hexagonal boron nitride (hBN) is a highly stable, large band gap 2D material that has found use not only as a substrate, barrier and encapsulant,^1–3^ but is also emerging as a platform for solid-state spin-photon and quantum sensing technologies.^4–6^ Catalytically enhanced chemical vapour deposition (CVD) on transition metal substrates such as Ni is the most promising route to scalable hBN film manufacturing.^7–14^ A major challenge for the research field and the industrial uptake of these ultra-thin films remains facile, non-destructive characterisation, such as assessing their structure and standardising their quality. This includes characterisation of the in-plane texture and grain boundary structure in monolayers and the out-of-plane stacking and stacking domain reconstruction of two or more such monolayers. Such a film structure significantly influences mechanical, chemical and opto-electronic properties^15–18^ and is a unique fingerprint of CVD nucleation, domain expansion and domain merger kinetics.^7–11^ Characterisation is often limited to imaging of “best-in-show” film areas after preparing a suspended film or transfer to suitable imaging substrates. However, for industrial scale-up, it is critical to be able to characterise hBN directly on the growth substrate and to capture and devise approaches to quantitatively compare film heterogeneity and polycrystallinity including the in-plane domain texture, adlayers, and relative stacking. Resolving nm-scale structural features and heterogeneity over cm-diameter area films requires characterisation to bridge traditional size-scale limitations. A further challenge is that mono/few-layer film samples are highly sensitive to a plethora of extrinsic effects including exposure to environmental conditions. Changes to the sample can therefore occur after transfer or storage in air and as a result of standard preparation steps, such as annealing, encapsulation or changes in the substrate.

Surface science studies of the hBN/Ni(111) system by low-energy electron microscopy (LEEM) and photoemission electron microscopy (PEEM) have reported both epitaxial and misaligned monolayer hBN domains,^19–21^ where the more strongly coupled epitaxial hBN domains show brighter contrast in PEEM associated with work function (WF) lowering on the order of 0.7 eV.^22^ However, widespread use of LEEM and PEEM is limited as these are specialised techniques,^23^ which require absolute surface planarity and cleanliness and typically demand an ultra-high vacuum environment. Transmission electron microscopy (TEM) analysis of CVD hBN monolayer films has shown that in addition to atomically stitched domain boundaries (DBs) overlapping DBs can also form, which are composed of bilayer regions.^24^ TEM has also been widely used to investigate the nature of point defects and edge terminations in hBN monolayers.^25^ However, TEM imaging requires preparation of a fully suspended sample, where key information on the interaction between the substrate and the hBN is lost.^26^ Cross-sectional TEM imaging has revealed the presence of a Ni_23_B_6_ interlayer between epitaxial single-crystal hexagonal boron nitride multilayers on Ni(111),^27^ but this requires extraction of a pristine, atomically thin slice at the surface, and the field of view is very limited (typically a few tens of nanometres). While we highlight the challenges of electron microscopy-based techniques, the inherent nature of atomically thin films and the need for simple, rapid fingerprinting across different size scales pose challenges for any current individual characterisation method, including scanning probe techniques.

Scanning electron microscopy (SEM) is a widely available, versatile, non-destructive technique, with nanometre-scale spatial resolution and can also bridge length scales to analyse centimetre-wide regions of interest. It is highly surface-sensitive, yet compatible with different substrates, and tolerant of sample roughness and imperfections (*i.e.* no need for meticulous surface preparation). SEM detects secondary electrons (SEs) that have been emitted from the sample on exposure to the electron probe. Conventionally, these secondary or low energy electrons are assigned to three categories. SE1 electrons are generated by the primary e-beam at the sample surface, SE2 electrons are generated at the sample surface by backscattered electrons (BSE) from within the sample, while SE3 electrons are generated at the surface of SEM components by BSE from the sample.^28^ A typical in-lens (or through-lens) SE detector signal is dominated by SE1 and SE2, whereas for a side-mounted Everhart–Thornley (ET) SE detector SE3 electrons are also important.

For an island of 2D materials supported on a substrate, such as graphene on Cu or hBN on Ni, the dominant SE signals are thought to be SE1 from the 2D material surface, SE2 from the 2D material surface generated by the substrate BSE, and SE1/SE2 emitted from the substrate but attenuated by the 2D layer.^29^ For metal substrates where there is no sample charging and for which the variation of BSE yield with primary e-beam energy is negligible, the typical SE contrast observed is for the area where the 2D material is present to appear darker than the bare metal.^30^ Thicker multi-layer graphene regions will also appear darker than thinner or monolayer areas, with optimum SE emissions achieved at low acceleration voltages.^31^ SE contrast for graphene on SiO_2_ has been found to increase linearly with thickness for 4 to 12 layer thick graphene,^32^ with anomalously high contrast seen for few-layer samples due to their decreased WF compared to graphite. Such quantitative assessment of SE contrast has enabled WF extrapolation of 2D materials with a precision of better than 10 meV.^33^ Furthermore, *a priori* knowledge of the specimen WF and thickness then allows 2D layer-thickness determination from SE contrast.^31^

Nonetheless, SE contrast in SEM is often challenging to interpret and depends on the detector configuration, system settings and specimen factors including differential surface charging. A key factor is the precise electron channelling conditions, which are determined by the accelerating voltage, sample tilt, the crystallography of the 2D material and the crystallographic orientation of the substrate. In particular, the SE2/SE3 yield will depend on the channelling in the underlying substrate. For example, the SE contrast of graphene on Cu has shown an inversion from dark to light contrast as a function of the sample tilt but only for some grain orientations. Tilting the sample changes the electron channelling conditions, as well as increasing the yield of SE3.^29^ For bilayer and thicker 2D materials, the stacking of the 2D crystal also affects the electron channelling conditions and hence the SE contrast. SEM imaging with an ET detector has revealed reconstructed domains of different crystal stacking in twisted graphene bilayers while similar twist domains have been studied in transition metal dichalcogenide (TMD) bilayers using both ET and in-lens detectors.^34–36^ For twisted bilayer TMD domains on standard substrates in the absence of sample tilt, similar contrast was observed for both BSE and SE detectors indicating that the SE signal is dominated by SE2. Differences in bilayer crystal stacking could be detected in twisted samples both at the top-most surface and when present as buried layers, enabling imaging of encapsulated structures.^35,36^

The SE contrast in SEM is also sensitive to the local coupling between the 2D material and the substrate. This is important as when imaging 2D CVD island samples on bare metal surfaces, neither the metal surface nor the metal-2D material interface might be pristine, but both can be affected by adsorbents, intercalants, reconstructions and precipitates linked to the growth kinetics and all post-growth exposures.^37–39^ The SEM contrast for graphene on Cu has been shown to have a strong dependence on the level of surface oxidation of the underlying substrate, which in turn depends on how strongly the graphene couples to the Cu.^40^ The Cu oxidation can lead to decoupling and an increase in the WF of the overall stack, lowering SE2 emission and subsequently mitigating contrast.^41^ The coupling of 2D layers to a metal support is highly dependent on their relative crystal orientations and can lead to notable changes in properties, with for instance hybridisation effects on Ni(111) causing epitaxial hBN to change from a wide-bandgap semiconductor to a metal-like band structure.^42^ For in-lens SE imaging of CVD hBN on Pt and Fe a near complete loss of SE contrast of isolated hBN monolayer islands has been observed upon prolonged ambient air exposure, likely due to the build-up of surface contaminants and intercalation of species.^9,39^

We focus here on SEM characterisation of scalable CVD of monolayer hBN on epitaxial Ni(111) wafers and demonstrate plan-view mapping of the complex in-plane crystallographic hBN texture including the nature of DBs and (reconstructed) stacking order in bilayer regions. We employ density functional theory (DFT) to model the hBN-Ni interface structures and calculate WF differences for experimentally observed interface reconstructions. Combined with data on hBN domain shape and alignment, we show that a self-consistent SE contrast assignment is possible. The primary assignment of in-plane hBN domain orientation also enables us to analyse the local DB structure and show that the nature of DBs strongly depends on the relative orientation of the two hBN domains. Specifically, we find overlapping AA′ stacked DBs form when two different epitaxial domains meet and reconstructed/mosaic domains are observed where epitaxial domains meet non-epitaxial domains.

## Experimental methods

### hBN CVD on Ni(111) growth

Monolayer hBN growth was performed using methods introduced in previous studies.^11,43^ Briefly, Ni(111) substrates were prepared by sputtering 500 nm of Ni on *c*-plane sapphire (Roditi International) using an AJA ATC sputtering system at 500 °C. hBN growth was performed using an Aixtron BM system with a pyrolytic BN coated graphite heater, where the substrates were heated to 950 °C in 3.5 mbar H_2_ and annealed for 30 min, followed by hBN growth with partial gas pressures of 7 × 10^−4^ mbar B_3_N_3_H_6_, 5 × 10^−2^ mbar NH_3_, 3.5 mbar H_2_ for 15 min for hBN domains, or 30–40 min for high hBN coverage films.

### SEM characterization

**Fig. 1:**
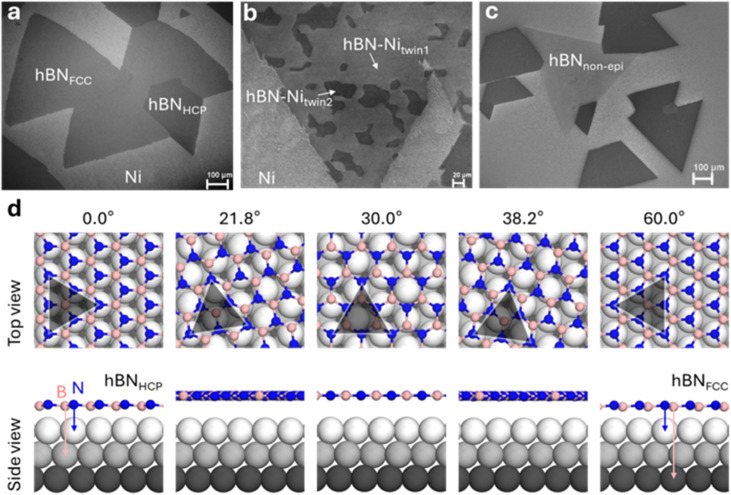
Contrast of hBN domains on Ni films. (a) Aligned epitaxial hBN domains on a twin-free Ni(111) single crystal. (b) A single crystal hBN domain crossing multiple twinned Ni(111) grains without changing its orientation and also exhibiting strong contrast on different Ni(111) twinned grains. (c) A non-epitaxial hBN domain rotated to the epitaxial domains by an arbitrary angle showing lighter contrast. (d) A selection of calculated structures for hBN on Ni(111) with different angles, along with a side view of the structure. 0° is the “HCP” epitaxial configuration and 60° is the “FCC” configuration, as discussed in the main text.

We used a Zeiss Gemini 300 field-emission SEM with standard in-lens and ET detectors and a vacuum base pressure <5 × 10^−6^ mbar. We explored a wide range of imaging conditions, including variations of the accelerating voltage from 3 to 10 kV (see ESI Fig. S14†), working distances from 3 mm to 17.5 mm, apertures from 30 μm to 120 μm and electron currents of 0.1–1 nA. We find that medium to low magnification (20–500×) are beneficial for reducing carbon writing on top of the samples. We observe that SE contrast is dependent on the relative hBN/Ni(111) alignment, which is most clearly visible using the in-lens detector for typical imaging conditions (5 kV, aperture of 60 or 120 μm, and 5 mm working distance). All samples were either imaged immediately after CVD and a brief air exposure due to sample transfer or within a period of 2 hours of air exposure after growth. We find that a significant change can occur for prolonged ambient air exposure (see ESI Fig. S13 and S16†), causing some samples to almost completely lose SE contrast (ESI Fig. S13†). We observe a contrast reversal for the in-lens detector depending on imaging conditions (see ESI Fig. S5 and S16†). Such in-lens detection phenomena can be highly column specific depending on signal electron filtering, detector position and experimental imaging conditions.^44,45^ Unlike to SiO_2_ support,^32^ we expect no significant effect due to differential surface charging for closely coupled hBN domains on metallic Ni. Thus, to map the relative SE contrast between differently aligned hBN domains and Ni(111), we consistently used fixed in-lens imaging conditions. The SEM images showed a high prevalence of roughly triangular shaped hBN domains with their edges aligned along directions corresponding to multiples of 60° relative to each other (see Fig. 1a). To measure the relative rotation angle for facetted hBN domains that did not conform to one of these preferred rotation angles, the angle between two lines was measured using ImageJ: one line being the edge of a 0° rotated triangle and the other being one of the edges of the misaligned domain (only one edge was measured as such domains are typically highly equilateral). The results were presented modulo 60° due to the three-fold symmetry.

### Image processing

SEM images were imported into MATLAB as 2D matrices, followed by applying a predefined 2D averaging filter. Filter size depended on the resolution of the image and feature sizes (*e.g.* 4 × 4 pixels). A histogram of the grayscale image intensity was then generated. A Voigt function of the form *y* = *y*_0_ + (*f* × *g*) was used, where (*f* × *g*) is a convolution of Lorentzian and Gaussian functions, respectively: 
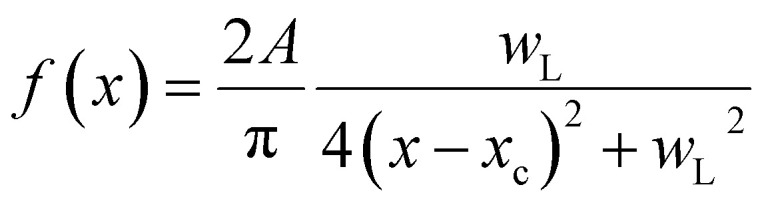
 and 
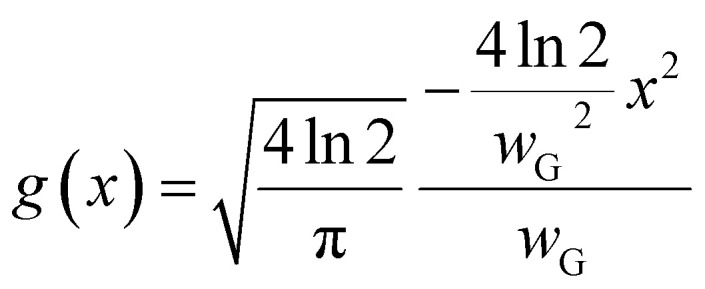
, where *y*_0_, *x*_c_, *A*, *w*_G_, and *w*_L_ are fitting parameters. The number of Voigt functions, the initial fitting parameters, and the constraints on the fitting parameters were selected manually. After fitting the functions, the accuracy of the fit was checked manually, and regions (grayscale intensity boundaries) where one function was above the others were assigned. White, red, green and blue channels were generated by assigning RGB triplets of [1 1 1], [1 0 0], [0 1 0], and [0 0 1] for the bare Ni, hBN_HCP_, hBN_FCC_, and hBN_bilayer_ grayscale intensity regions and combining them into a texture map with a multidimensional concatenate array function.

### DFT calculation

Spin-polarized DFT calculations were performed with CASTEP.^46^ The PBE functional of the generalized gradient approximation (GGA) was adopted to describe the exchange correlation effects.^47^ The C19 on-the-fly generated ultrasoft pseudopotentials were used to describe the ion–electron interactions. The energy cutoff for the plane wave basis set was chosen to be 700 eV. The Monkhorst Pack grid was adopted to integrate the Brillouin zone, with a *k*-point spacing of 0.03 2π Å^−1^.^48^ The van der Waals interactions were taken into account using Grimme's semi-empirical dispersion correction scheme.^49^ Self-consistent dipole correction was applied in the direction perpendicular to the surface. The convergence tolerance for the electronic minimization was set to 0.5 × 10^−6^ eV per atom, while the tolerance for the geometry optimization was set to 0.5 × 10^−5^ eV per atom for the energy, 0.1 × 10^−1^ eV Å^−1^ for the force, 0.5 × 10^−3^ Å for the displacement, and 0.2 × 10^−1^ GPa for the stress.

### hBN on the Ni(111) model

We first fully optimized the structures of Ni metal and monolayer hBN. Using the optimized crystal structures, a Ni(111) slab model containing three Ni atomic layers with a vacuum of 15 Å was constructed. The bottommost Ni atomic layer was fixed in all calculations. From these unit cells, we constructed models of hBN on the Ni(111) surface with various orientation angles. Due to the different lattice parameters of hBN and Ni(111), supercell models were constructed in a manner that minimizes strain, following the approach used in Kosma and Krasheninnikov's work.^50^ Based on the unit cell lattice vectors of Ni(111) (**a**_Ni(111)_ and **b**_Ni(111)_) and h-BN (**a**_hBN_ and **b**_hBN_), supercell lattice vectors were constructed as*n*_Ni(111)_ × **a**_Ni(111)_ + *m*_Ni(111)_ × **b**_Ni(111)_*n*_hBN_ × **a**_hBN_ + *m*_hBN_ × **b**_hBN_.

The second lattice vectors are always oriented at a 120° angle.*o*_Ni(111)_ × **a**_Ni(111)_ + *p*_Ni(111)_ × **b**_Ni(111)_*o*_hBN_ × **a**_hBN_ + *p*_hBN_ × **b**_hBN_.

We then search for a set of integers (*n*, *m*, *o*, and *p*) such that the magnitude of the supercell lattice vectors in Ni(111) and hBN approximately match.‖*n*_Ni(111)_ × **a**_Ni(111)_ + *m*_Ni(111)_ × **b**_Ni(111)_‖ ≈ ‖*n*_hBN_ × **a**_hBN_ + *m*_hBN_ × **b**_hBN_‖

The smallest supercells with less than 3% strains were selected for calculation. Additionally, structures with rotation angles up to 60° were taken into account, considering the symmetry of Ni(111) and hBN. The resulting structures are summarized in ESI Table 1 and Fig. S4.†

### Potential energy surface analysis

To systematically construct and explore the potential energy surface for different stacking of hBN bilayers, we employ the ab-plane scan approach. Starting from the AA′ stacked bilayer, various stacking configurations are generated by applying a displacement vector to the topmost layer while keeping the bottom layer or Ni substrate fixed. Stacking configurations resulting from ab-plane displacements were sampled using a 16 × 16 grid with uniform spacing, and DFT single point energy calculations were carried out.

## Results and discussion

### Understanding SE contrast due to monolayer hBN domain rotation on Ni(111)

We begin by considering the SEM contrast of isolated hBN monolayer domains synthesized on single crystal Ni(111), *i.e.* early-stage growth prior to domain merger and continuous film formation. We confirm the monolayer nature of hBN by Raman spectroscopy (ESI Fig. S2; † with hBN transferred to SiO_2_/Si). In-lens SEM images for the given conditions show the hBN domains as triangles or truncated triangles of lower SE image intensity than the substrate (darker) with >100 μm lateral dimensions,^51^ which are highly aligned with each other, as shown in Fig. 1a. As the rotation angle of the triangles reflects the crystallography of the hBN basal plane, the alignment of the triangles demonstrates their hetero-epitaxy with the substrate. This epitaxy is favoured due to the small lattice mismatch between the hBN basal plane and the Ni(111) substrate (0.5%) creating strong hBN(epi)–Ni interactions. The detailed hBN domain morphology will depend on the CVD conditions, substrate steps, and its crystallography, often creating deviations from a perfect triangular shape.^52^ The thermodynamically preferential epitaxial structure is considered to be one where the N atoms are directly above the top row of the Ni(111) atoms, while the B atoms occupy the “FCC sites” or simply are above the 3rd row of Ni atoms, with a reported atomic corrugation in the hBN layer (Fig. 1d: 60° structure).^53,54^ There is also a different epitaxial configuration where the B atoms reside on the “HCP” Ni(111) sites or simply above the 2nd row of Ni atoms (Fig. 1d: 0° structure). Macroscopically, these two epitaxial hBN domains will appear as 180° rotated triangles, often termed “anti-parallel”.^55^ Previous studies have shown that the FCC configuration is only slightly more stable than the HCP one (0.04 eV difference in the formation energy),^51^ and hence it is common to observe the two anti-parallel hBN domain orientations on perfect Ni(111) single crystal substrates.^19^ The situation is slightly complicated as Ni(111) surfaces are prone to twinning, and the presence of twins will also cause anti-parallel hBN domain orientations when the hBN domains nucleate in one region of the Ni film and cross multiple twinned Ni(111) regions [EBSD data to confirm occasional Ni(111) twinning are shown in ESI Fig. S1†]. We find that under our SEM conditions using an in-lens SE detector (Experimental methods) there is a distinctive difference in SE contrast between these anti-parallel epitaxial hBN domains. This difference can be seen when there are FCC and HCP configured hBN domains (Fig. 1a) or when the Ni(111) film is twinned (Fig. 1b).

**Fig. 2:**
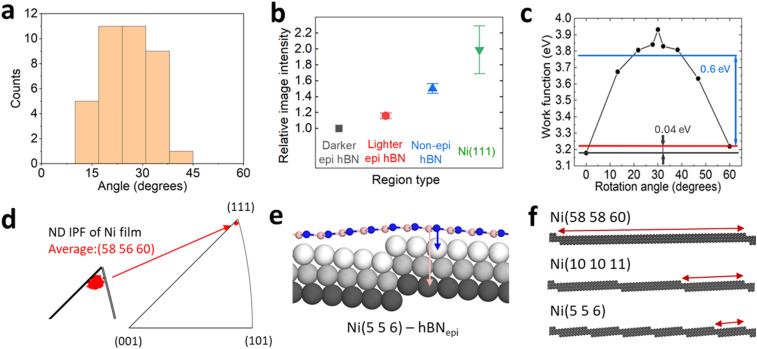
Analysis of hBN domains and the Ni(111) surface. (a) Histogram showing the distribution of the rotation angles for non-epitaxial hBN domains (excluding epitaxial angles of 0° and 60°). (b) Relative average pixel intensity of the in-lens SEM signal for the lighter epitaxial hBN, non-epitaxial hBN and bare Ni(111) substrate normalized to the darker epitaxial hBN domain pixel intensity (0° or “HCP” configuration). The larger variability of the bare Ni(111) value is likely affected by the presence of small segregated hBN domains in some samples, with the true value for a bare metal surface thus being closer to the top of the error bar (around 2.2). (c) Calculated work function for different rotation angles of hBN on Ni(111) surfaces, showing that the FCC epitaxial domains are 40 meV higher than the HCP epitaxial domains and non-epitaxial domains are around 600 meV higher than this. (d) The ND IPF plot of orientations normal to the sample surface measured from a high-resolution EBSD scan of a large area Ni film (200 μm × 200 μm). The centre is close to a crystallographic Ni(58 56 60) zone axis with some radial distribution around it due to mosaic spread in the film. (e) An example structure of the epitaxial hBN-Ni surface for a step on a more representative Ni(556) surface. (f) Examples of other surface terminations showing the occurrence of atomic steps.

We also observe the formation of a third, less common hBN configuration, which manifests itself as a macroscopic triangle that is rotated by an arbitrary angle relative to the base of the epitaxial triangles (modulo 60°, Experimental methods), as shown in Fig. 1c. These rotated hBN domains appear much lighter, closer to the greyscale of bare Ni. Also, we find that these hBN domains mostly have a perfect triangular shape without being affected by the substrate crystallography, unlike the epitaxial domains. These observations are consistent with such hBN domains not being epitaxial and having weaker interactions with the substrate. We analyse multiple samples and images (ESI Fig. S3†) and find that the rotation angles for these lighter hBN domains vary between 15° and 45° (Fig. 2a). This is in good agreement with prior work on non-epitaxial hBN domains using LEEM.^22^ We statistically analyse multiple SEM images and normalize the image intensity to the darkest hBN domain pixel intensity, as shown in Fig. 2b. The relative pixel intensity between the bare Ni(111) surface, non-epitaxial hBN domains, lighter epitaxial hBN, and darker epitaxial hBN domains is approximately 2 : 1.5 : 1.2 : 1. The range for the pristine Ni(111) relative image intensity is comparatively large due to the possibility of small (<1 μm, ESI Fig. S7†) hBN domains on the surface caused by hBN precipitation upon cooling or bulk-mediated processes,^10^ with the bare Ni(111) value likely to be closer to the higher end of the error bar (around 2.2).

When considering the SE contrast of monolayer 2D materials on metal substrates, the SE generation and transport are dominated by the substrate, but the SE escape probability is governed by the WF of the 2D material–metal interface, which can drastically change the escape probability of BSEs that go on to generate SE2 as well as the angular distribution of SEs being emitted from the surface.^56,57^ To calculate the differences in the WF for these different structures, we consider 7 possible crystal structures of hBN on Ni(111) with different rotation angles and perform DFT calculations (Fig. 1d, ESI Table 1, ESI Fig. S4,† Experimental methods). The values of the calculated WFs are shown in Fig. 2c for different rotation angles of hBN on the substrate, with the two epitaxial domains being at 0° (“HCP” type) and 60° (“FCC” type). The calculated WF difference between the epitaxial and non-epitaxial domains (average for the set) is around 600 meV. This agrees well with previous literature on the Ni(111)–hBN system, which reported a calculated WF difference of 700 meV, also confirmed experimentally with *I*–*V* LEEM.^22,58^ Our calculations also show that the two epitaxial domains (FCC and HCP types) on Ni(111) have a WF difference of around 40 meV, with FCC having a higher WF. For our imaging conditions (Experimental methods), the non-epitaxial domains with a higher WF have the brightest SE signal compared to the bare Ni substrate and the FCC epitaxial domains having the next highest SE signal, while the darkest domains are HCP epitaxially aligned. DFT calculations also show that the total energy of misaligned hBN domains (averaging over 20° to 40° rotation angles) is 110.9 meV higher than for the epitaxial domains.^22^ This may explain why 0° to 15° and 45° to 60° rotations (Methods) are not seen in our experimental results: the non-epitaxial hBN nuclei close to 0° or 60° are able to re-arrange themselves to align to become an epitaxial hBN nucleus in the early stages of the growth driven by the lower energy configuration.

To better understand the nature of the “Ni(111)” surface, we perform large-area EBSD mapping (200 μm × 200 μm) of our samples and plot the distribution of Ni surface orientations on a normal direction inverse pole figure (ND IPF, Fig. 2d). For our sample, the average is centred on a Ni(58 56 60) zone axis, with a distribution of other surfaces around it, for example Ni(5 5 6). This misorientation of the surface is not uncommon and what is often for simplicity referred to as a “Ni(111)” substrate may not be perfectly flat but have a distribution of surface terminations around some higher-index orientation. The precise misorientation is likely determined by complex Ni film growth processes and sapphire substrate-Ni interactions, while the width of the distribution is determined by the mosaic spread within the single crystal film. The small offset from perfect Ni(111) is accommodated by the presence of atomic steps interfacing atomically flat Ni(111) terraces of various lengths (Fig. 2f). Such small vicinal misorientation of realistic metallic film surfaces grown on sapphire or other substrates is rarely discussed in the literature and its detailed effects on 2D material growth are not known. Our previous overview of the literature pointed,^59^ for example, to significant discrepancies regarding reports of hBN growth on Cu(111), where some studies indicated the inability to grow hBN single crystals on Cu(111),^60^ while others achieved 99.6% hBN domain alignment.^61^ Our analysis of the hBN–Ni system shows that approximating to the closest low-index orientation may not be sufficient to achieve comparable results between different reports in the literature. Instead, the average orientation and mosaic spread of the metal catalyst film should also be assessed using EBSD, as shown in Fig. 2d, to better understand the CVD growth behaviour.

**Fig. 3:**
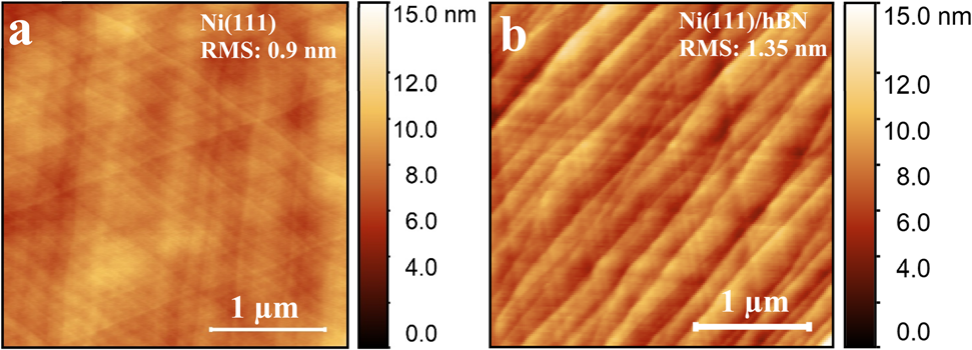
(a and b) AFM scans of the Ni(111) surface before and after hBN growth by CVD showing terrace formation. Root mean squared roughness values are inset on the images.

Furthermore, as hBN domains grow, they significantly change the Ni surface topography, as shown in Fig. 3 and ESI Fig. S15,† adding multi-atom steps and extended terraces with widths of hundreds of nanometres.^62^ Such faceting phenomena arise due to a delicate balance between the anisotropic interfacial surface energy minimization and the 2D material strain.^63,64^ We find that the surface roughness of the Ni(111) surface increases from around 0.9 nm to 1.35 nm after hBN CVD growth, and it is possible that this could also have an effect on the WF value, for example, by causing different Ni coupling over rougher surfaces where hBN regions are suspended (Fig. 2e). To understand the potential role of a metal surface with a higher index zone axis on the WF contrast, we create a Ni(5 5 6)–hBN surface (Fig. 2e and ESI Fig. S6†), which is within our measured Ni orientations, but is also sufficiently simple to perform DFT calculations, thus providing a better representation of realistic Ni surfaces grown on sapphire. With this structure, we obtain a WF difference between the two epitaxial orientations of 50 meV, slightly higher than that on the perfectly flat Ni(111) surfaces (40 meV). We attribute the increase to the influence of the atomic Ni steps. For comparison, we can also consider several related surfaces as shown in Fig. 2f to visualize the occurrence of atomic steps across various Ni surfaces present in the sample, suggesting that the WF value on realistic, kinked surfaces could be more than 50 meV. We note that we do not observe a difference in SE contrast between differently oriented non-epitaxial hBN domains. We suggest that this is because the coupling to the Ni is very weak for these, facilitating easier gas penetration at the interface. This is consistent with the observed loss of SE contrast upon air exposure.

### Understanding SE contrast of hBN domain boundaries and bilayer regions

**Fig. 4:**
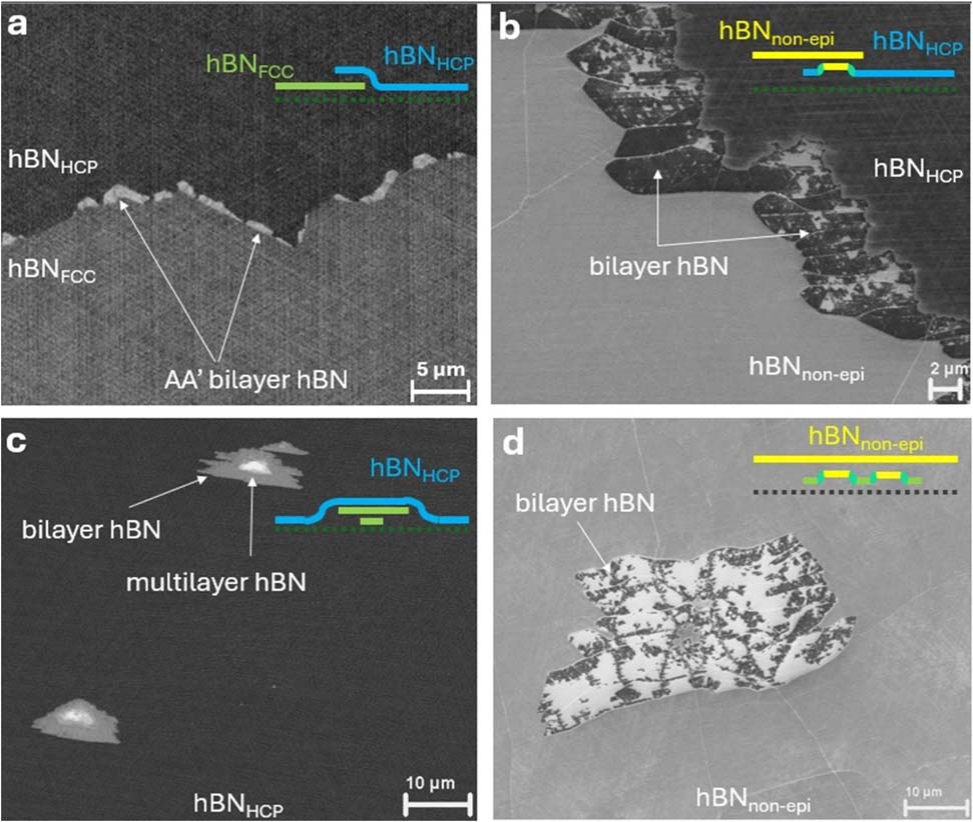
Other types of SE contrast in SEM images of hBN on Ni(111) films. (a) Interface between a FCC epitaxial domain with an HCP epitaxial domain, resulting in the formation of a bilayer overlap region (light). (b) Interface between an epitaxial HCP domain and a non-epitaxial domain resulting in an overlapping, darker bilayer with lighter patches. (c) Formation of hBN adlayers within a HCP epitaxial domain at the later stages of film formation (bilayer triangles with smaller multilayer triangles in the middle). (d) Formation of a bilayer with both lighter and darker contrast underneath a non-epitaxial hBN domain.

**Fig. 5:**
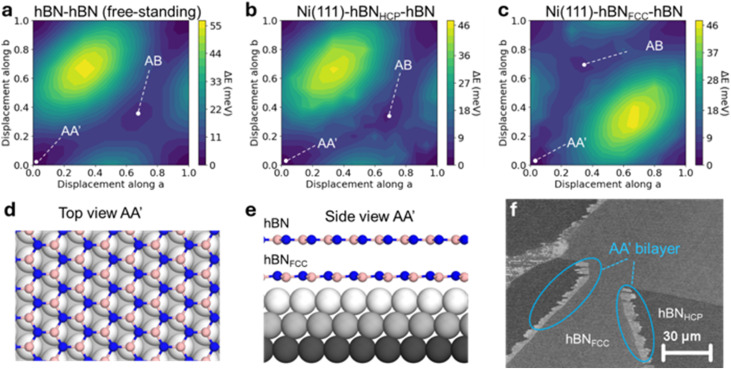
Analysis of bilayer configuration between the two epitaxial hBN monolayers. (a–c) Potential energy surfaces of bilayer hBN: free-standing, Ni–hBN_HCP_–hBN, and Ni–hBN_FCC_–hBN, respectively. The lowest energy configuration is attributed to AA′ stacking in the Ni–hBN_FCC_–hBN structure. (d and e) Top and side views, respectively, for the calculated lowest energy structure (Ni–hBN_FCC_–hBN with an AA′ stacked hBN bilayer). (f) A SEM image of the interface between two epitaxial hBN domains. Notably, the lighter hBN region (FCC-type) grows into the darker region (HCP-type), confirming that the interaction between Ni and hBN_FCC_ is stronger than between Ni and hBN_HCP_ and that the most likely configuration is as in (d and e).

Having understood SE contrast differences for monolayer hBN domains of a given orientation, we proceed to study samples exposed to longer CVD growth times, in which different hBN domains start to meet and interact. In such samples, we discover a number of different film evolution scenarios. We first investigate what happens when two epitaxial hBN domains meet. If the two domains are of the same orientation, we do not observe any variation in SE contrast along the potential boundary of their merging, indicating the absence of overlapping or wrinkles and potentially suggesting seamless stitching (ESI Fig. S7†). However, where two anti-parallel epitaxial domains meet, we always observe SE contrast variation indicative of overlaps suggesting that one domain grows under the other, as shown in Fig. 4a (and ESI Fig. S7†). Such overlapping hBN DBs have been analysed by TEM before but only for nm-sized fields of view.^65^ Our SEM approach allows observation of the morphology of such overlapping boundaries over mm scales and without transfer or elaborate sample preparation. The bilayer hBN overlap regions appear brighter than the surrounding monolayer for our SEM imaging. We find that the lighter epitaxial hBN region (“FCC” type) exclusively spreads into the darker epitaxial hBN region (“HCP” type)), albeit relatively slowly, as shown in Fig. 5f and ESI Fig. S8b.† Our observation confirms that the “FCC”-type hBN is more strongly attached to Ni and therefore grows underneath the “HCP”-type monolayer hBN, in agreement with previous DFT calculations.^51^ This understanding potentially opens up new routes to produce bilayer hBN regions with controlled stacking, for example for spintronic applications,^66^ by utilizing seed “FCC” layers that would grow into regions of a predominantly “HCP” hBN on Ni. We have observed such Ni-hBN_FCC_-hBN stacks with lateral sizes of up to 20 μm for extended growth times (Methods, ESI Figs. S8b, S11, S12†).

From simple geometrical considerations, the type of stacking in the bilayer between the two epitaxial domains could be of the AB2′ type; however, the Ni–hBN–hBN interaction also needs to be considered. We perform DFT calculations of such structures between two anti-parallel epi hBN domains for free-standing bilayer hBN, Ni–hBN_HCP_–hBN_FCC_ and Ni–hBN_FCC_–hBN_HCP_ (see the Experimental methods). Potential energy surfaces relative to displacements along the lattice unit vectors are shown in Fig. 5a–c. Irrespective of the presence of Ni, we find that the AA′ configuration (N over B and B over N) is the lowest energy configuration, with the Ni-hBN(“FCC”)-hBN being preferred overall (ESI Table 2, Fig. S8a†). This is in line with previous literature on the stability of the FCC-type layer^51^ and that the AA′ bilayer stacking is energetically favourable under CVD conditions used for high-quality hBN growth.^18,27,67^ The top and side atomistic profiles of this configuration are shown in Fig. 5d and e. We also perform DFT calculations of the WF of such material stacks and find that the Ni–hBN_FCC_–hBN stack has a WF of 3.33 eV that is significantly lower than the freestanding bilayer hBN or bulk hBN WF of around 5.7 eV (ESI Table 2, Methods). This WF is 80 meV higher than that of the monolayer FCC epitaxial domains. However, we cannot compare monolayer and bi-layer regions using WF alone, because the SE yield is likely affected by the presence of a second hBN layer. We therefore attribute the lighter areas between antiparallel domains to bilayer hBN, likely with AA′ stacking, as seen in Fig. 5f.

A very different scenario occurs when epitaxial and non-epitaxial hBN domains meet, as shown in Fig. 4b and ESI Fig. S9.† Here, the resulting bilayer region is typically larger than the bilayer region between two epitaxial domains for the same growth time. For example, after 1 h growth time the overlap between epitaxial and non-epitaxial hBN has a width of around 30–50 μm, while the overlap between the two epitaxial domains is around 10 μm (Methods, ESI Fig. S12†). This is likely due to the weaker coupling of the non-epitaxial domains to Ni making it less energetically challenging for the epitaxial domain to continue to grow underneath it. At higher magnification (ESI Fig. S9b†), we observe small (100–1000 nm) triangular light and dark regions within such bilayer regions. Based on our previous discussion of relative SE intensity, we hypothesise that the light regions correspond to re-stacking of the hBN–hBN layers to locally adopt AA′ stacking with increased WF and a weaker attachment to the Ni layer, while the dark regions correspond to weaker turbostratic stacking between hBN layers, where the bottom layer is strongly attached to the Ni surface. Such local domain stacking reconstruction has been observed in marginally twisted multilayer hBN or multilayer TMDs, resulting in charge-polarized interfacial superlattices and ferroelectric domains,^68,69^ respectively. However, in our case, the interaction with Ni is prevalent, and the twist angles between the epi and non-epi monolayers (Fig. 2a) are quite large (15°–45°), resulting in the observed Ni–hBN–hBN local re-stacking between merging epi and non-epi domains.

Under optimized CVD growth conditions that promote a low nucleation density and large hBN domain sizes (*i.e.* slow growth at a low borazine partial pressure; see the Methods), it is uncommon to see multilayer formation and it is possible to obtain fully monolayer hBN films (excluding bilayers from overlapping DBs). However, occasionally in full coverage films or runs with higher borazine partial pressure, bilayer and multilayer hBN patches or adlayers grow. These grow catalytically underneath the existing hBN monolayer from sub-surface dissolved species.^27^Fig. 4c and d show typical adlayer examples for the cases of epitaxial hBN and non-epitaxial hBN. For the former, the bilayer region appears lighter (Fig. 4c), with a SE signal similar to the bilayer that appears when two anti-parallel epitaxial grains overlap. For the latter case of adlayer growth underneath non-epitaxial hBN regions (Fig. 4d), we observe a mix of dark and light regions indicating a mix of AA′ stacking in the hBN bilayer and epitaxial alignment between the Ni and the adjacent hBN monolayer (Fig. 4d). This is consistent with LEEM mapping on hBN on Ni that found AA′ stacked hBN bilayer formation underneath non-epitaxial monolayers, but did not find any underneath epi monolayers.^19^ These non-epitaxial adlayer regions are more frequent than the bilayers under the epitaxial regions (as seen in ESI Fig. S3b†) as expected due to the weaker coupling of the non-epitaxial domains to the Ni growth substrate, reducing the energy barrier against adlayer growth.

### SE contrast of the crystallographic texture and bilayer regions of high coverage hBN films

**Fig. 6:**
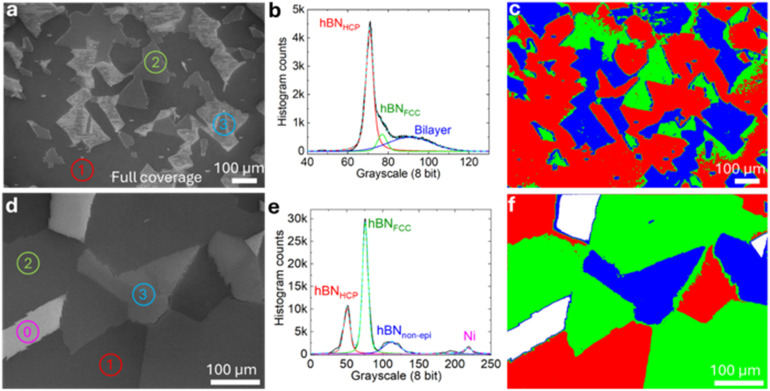
Analysis of SE contrast of hBN films on Ni(111). (a) An example of a continuous hBN film on Ni(111) consisting of (1) hBN_HCP_, (2) hBN_FCC_, and (3) hBN_non-epi_ bilayer and overlapping DB bilayer regions. There are also occasional multilayer patches (small white dots), which are not segmented separately but included in (3). (b) A histogram of pixel intensity for image (a) showing three fitted Voigt functions corresponding to the three typical hBN coverage regions. (c) A crystallographic texture map of the hBN coverage classification. (d–f) Similar image processing and reconstruction of a hBN film with incomplete coverage, where a further intensity classification is added corresponding to bare Ni surfaces (marked as (0) in (d) and represented by white in the texture map in (f)).

Based on the above insights, we proceed to interpret the complexity of full coverage CVD hBN films. Fig. 6a (and ESI Fig. S10–S12†) shows a rich variety of SE contrast features that we can now rationalise. The darkest regions, labelled as (1), are HCP-type epitaxial hBN with small adlayer patches visible as light spots. These regions interface with lighter regions, labelled as (2), which correspond to FCC-type epitaxial hBN. At boundaries where these regions meet, there are lighter regions, corresponding to AA′-stacked bilayers formed where the domains overlap. Because the contrast of bilayers is very similar to rotated monolayers, these regions together are labelled as (3). When the growth is performed for an extended time (see the Experimental methods), the epitaxial regions continue to grow underneath the non-epi regions and form continuous bilayers. Therefore, regions labelled as (3) in Fig. 6a are predominantly bilayer regions in this sample. Apart from identifying various regions, we can now create procedures for statistical analysis of large (mm-size) sample regions and this can be extended to wafer-scale mapping. To do this, we generate a histogram of the greyscale intensities (8 bit scale), which can be wholly fitted with 3 Voigt curves (see the Experimental methods), as shown in Fig. 6b. By selecting the regions from the mean of the Voigt curves to the cross-point between the curves, we can assign regions of different greyscale intensities to different types of hBN coverage. After such processing, a map with the detected regions can be generated, as shown in Fig. 6c. We calculate that in this sample the “HCP” type hBN monolayer covers 53% of the image, while the “FCC” type hBN monolayer covers 18%, and finally, the rotated hBN converted to bilayer and bilayer grain boundaries of different types cover 29%.

For samples with incomplete hBN coverage, a 4th Voigt curve needs to be added to the processing to account for the brightest signal from uncovered Ni surfaces, labelled (0) in Fig. 6d. We can then calibrate the image processing to identify 4 regions: the HCP epitaxial hBN domains, FCC epitaxial hBN domains, non-epitaxial hBN domains and bare Ni. After fitting 4 Voigt curves (Fig. 6e), it is possible to generate a map of the local distribution of hBN coverage, as shown in Fig. 6f. From this analysis, we conclude that the total hBN coverage is 93%, of which 26% is hBN_HCP_, 58% hBN_FCC_, and 16% hBN_non-epi_, respectively. This large-area statistical sample analysis could be readily automated and allows us to develop a quality metric for the CVD growth and rapidly quantify the crystallographic texture of hBN high coverage films over large areas. Such fast feedback loops can open up future CVD experimentation and optimization across the vast synthetic parameter space, influenced by a variety of factors, such as gas and precursor chemistries, flow geometries, substrate crystallography, substrate crystallinity (mosaic spread), substrate vicinal orientation, roughness or surface steps. The developed large area analysis using simple, rapid and non-destructive SEM imaging thus opens up powerful capabilities for quality control and process optimization.

## Conclusions

Atomic structure information is typically a realm of scanning tunnelling microscopy, TEM and other time consuming, expensive and cumbersome atomic-resolution techniques. Yet here we demonstrate that it is possible to fingerprint the atomic registry of hBN atoms on the Ni lattice, such as “FCC” and “HCP” configured epitaxial hBN domain types on Ni(111) or twisted non-epitaxial domains, with simple and fast SEM analysis. It is also possible to identify hBN bilayers and adlayers and propose their formation mechanisms including the likely stacking configuration for overlapping hBN growth at the boundary between two anti-parallel epitaxial domains or for epitaxial domain growth under existing non-epitaxial domains. While the hBN–Ni(111) system could be considered a simple model system with a low lattice mismatch and good catalytic ability of Ni, the system is experimentally challenging to optimise and we find a rich variety of structural features in typical full coverage CVD hBN films on Ni(111). The demonstrated crystallographic texture mapping opens new rapid and facile feedback capability for advanced CVD process development and in-line quality and reproducibility control.

## Conflicts of interest

There are no conflicts to declare.

## Supplementary Material

NA-007-D5NA00457H-s001

## Data Availability

The data supporting this article have been included as part of the ESI.†
