# Supplementary material for: Fast crystallographic texture mapping of atomically thin hBN films on Ni(111) using secondary electron contrast
Source: Nanoscale Adv. 2025 Jul 2;7(17):5262–72. doi: 10.1039/d5na00457h (PMC12278833; doi:10.1039/d5na00457h)
Supplement: NA-007-D5NA00457H-s001 [file NA-007-D5NA00457H-s001.pdf]

Supplementary Information (SI) for Nanoscale Advances.
This journal is © The Royal Society of Chemistry 2025

Fast Crystallographic Texture Mapping of Atomically-thin hBN Films on Ni(111) using Secondary Electron Contrast

Vitaliy Babenko[1], Se Hun Joo[2,3], Anastasia Krief[1], Evan Tillotson[4], Sarah J. Haigh[4], Chris J. Pickard[2,5], Stephan Hofmann[1,]*

[1] Department of Engineering, University of Cambridge, Cambridge CB3 0FA, United Kingdom.
[2] Department of Materials Science & Metallurgy, University of Cambridge, Cambridge CB3 0FS, United Kingdom.
[3] Department of Chemical and Biological Engineering, Sookmyung Women's University, Seoul 04310, Republic of Korea
[4] Department of Materials, University of Manchester, Manchester M13 9PL, United Kingdom.
[5]Advanced Institute for Materials Research, Tohoku University, Sendai, 980-8577, Japan

* sh315@cam.ac.uk

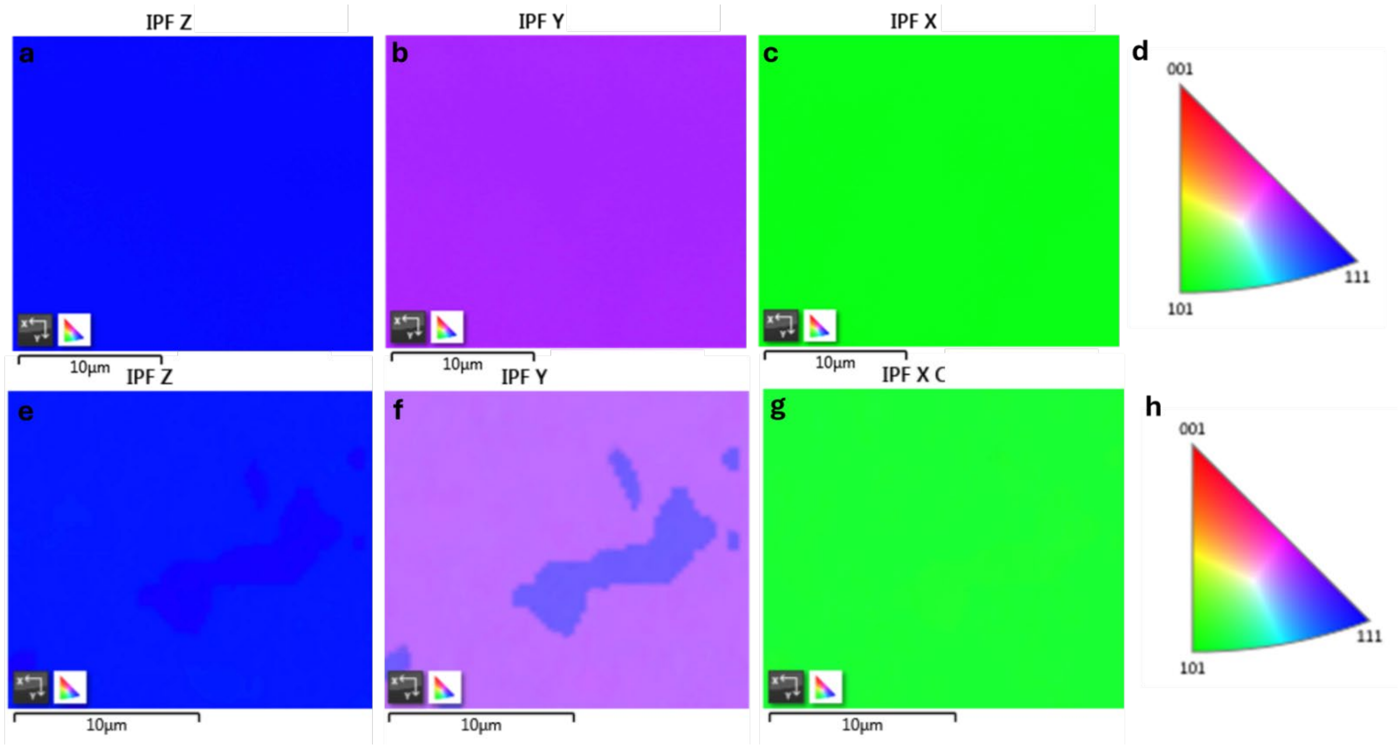

Fig. S1. (a-d) EBSD mapping of a Ni(111) region without any twin grains. (e-h) EBSD mapping of a Ni(111) film region with some twin grains visible from IPF Y.

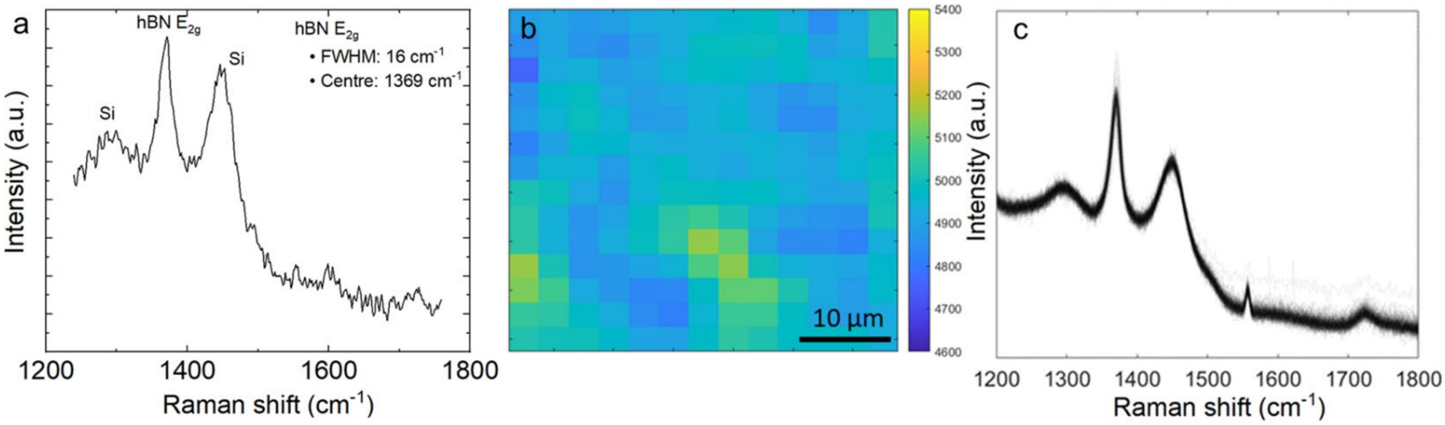

Fig. S2. (a) Raman signal of hBN grown on Ni(111) (see Methods) and transferred to a Si/SiO_2 substrate (285 nm) using a standard wet "etching" process with $FeCl_3$ and a polycarbonate support.[1] After subtracting the Si background, the position of the hBN peak and its width can be estimated as 1369 cm^{-1} and 16 cm^{-1} , respectively, consistent with monolayer hBN from prior reports.[1] (b) Raman intensity map (arbitrary units) of the E_{2g} peak at 1370 cm^{-1} of transferred hBN. (c) All Raman spectra from the map in (b) plotted with a 10% transparency showing the average spectrum pattern for the map, consistent with uniform monolayer hBN.

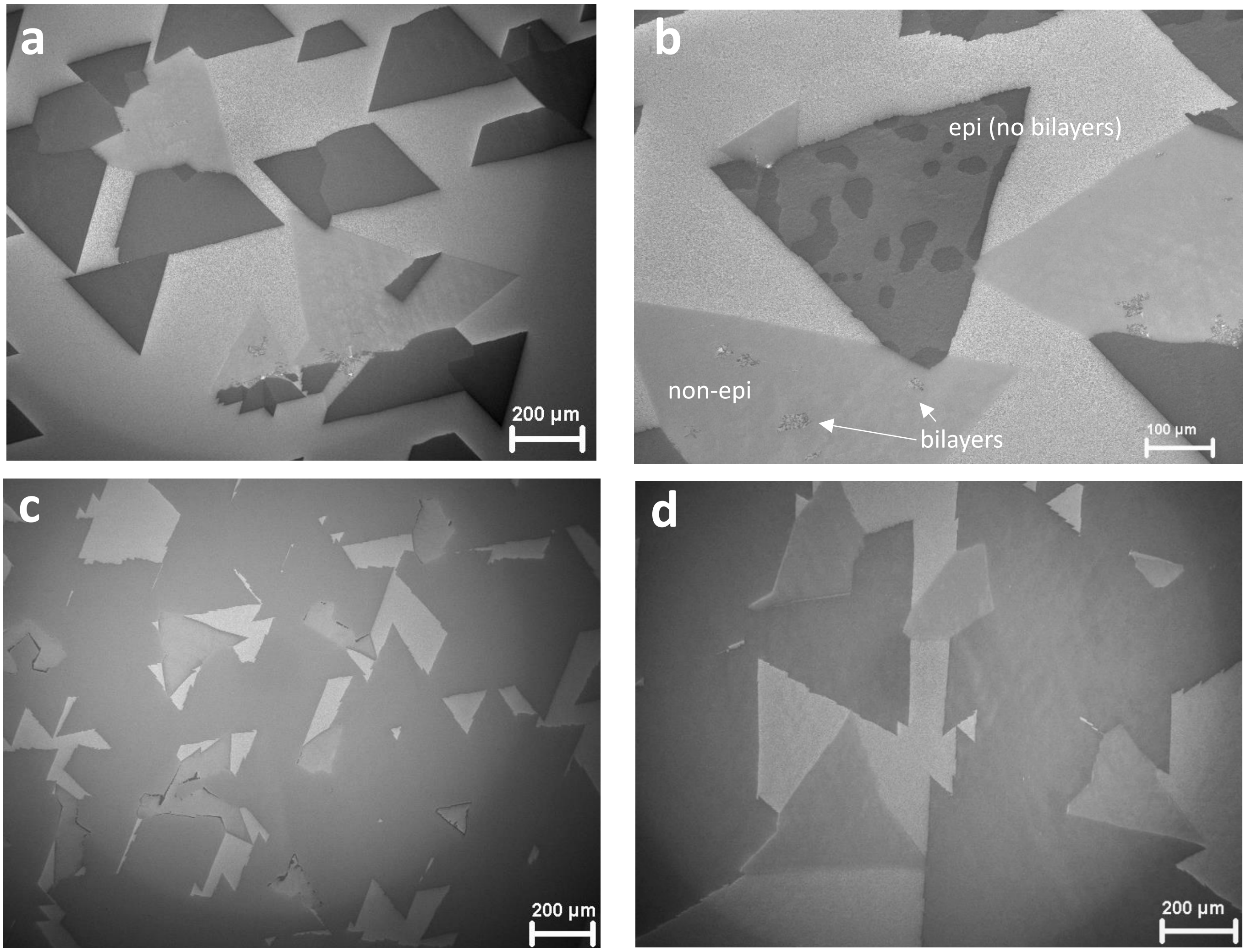

Fig. S3. SEM images (in-lens detector, 5 kV) of other examples of epitaxial and non-epitaxial hBN domains on Ni(111) used for the angle distribution calculation in Fig. 2a.

Angle (°)	Supercell basis vectors (*n*, *m*), (*o*, *p*)		Lattice parameter (Å)		Strain (%)
	Ni(111)	hBN	Ni(111)	hBN	
0.0	(1, 0), (0, 1)	(1, 0), (0, 1)	2.45	2.51	2.57
13.2	(5, 2), (-2, 3)	(5, 3), (-3, 2)	10.67	10.94	2.57
21.8	(3, 1), (-1, 2)	(3, 2), (-2, 1)	6.48	6.64	2.57
27.8	(4, 3), (-3, 1)	(3, 4), (-4, -1)	8.83	9.05	2.57
30.0	(6, 3), (-3, 3)	(5, 0), (0, 5)	12.72	12.55	1.31
32.2	(4, 1), (-1, 3)	(4, 3), (-3, 1)	8.83	9.05	2.57
38.2	(3, 2), (-2, 1)	(2, 3), (-3, -1)	6.48	6.64	2.57
46.8	(5, 3), (-3, 2)	(3, 5), (-5, -2)	10.67	10.94	2.57
60.0	(1, 0), (0, 1)	(1, 1), (-1, 0)	2.45	2.51	2.57

Table 1. Details of supercell models for hBN on Ni(111) with different rotation angles. The models were constructed using the optimized structures of Ni metal and monolayer hBN. The optimized lattice parameters of Ni metal are $a = b = c = 3.46$ Å. $\alpha = \beta = \gamma = 90°$. The unit cell parameters of Ni(111) are $a = b = 2.45$ Å, $c = 19.00$ Å. $\alpha = \beta = 90°$, and $\gamma = 120°$. For hBN, the optimized lattice parameters are $a = b = 2.51$ Å, $c = 15.00$ Å. $\alpha = \beta = 90°$, and $\gamma = 120°$.

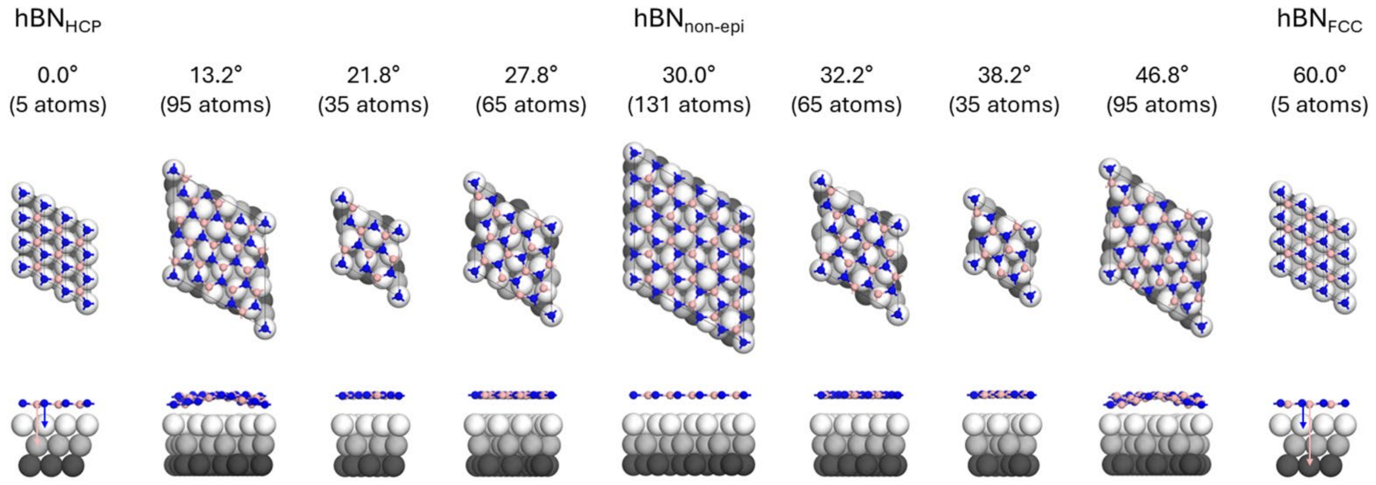

Fig. S4. Top and side views of optimized structures of hBN on Ni(111) with different rotation angles. 0° is the "HCP" epitaxial configuration, and 60° is the "FCC" configuration, as discussed in the main text. Boron atoms are represented in pink, and nitrogen atoms in blue. The Ni(111) substrate consists of three atomic layers, depicted in white, light grey, and dark grey.

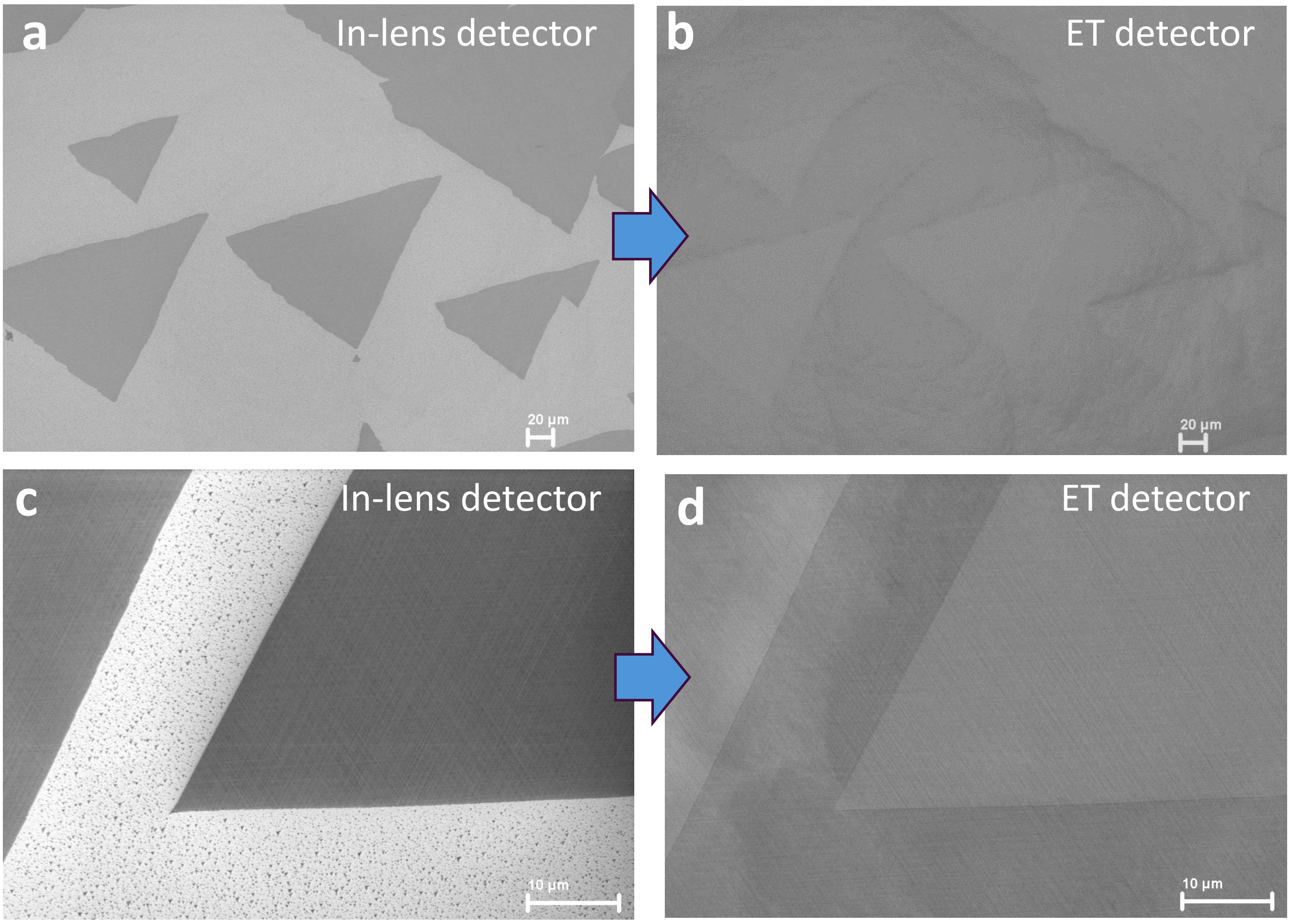

Fig. S5. Contrast reversal for hBN on Ni(111) between the in-lens and Everhart-Thornley (ET) detectors (5 kV, imaging conditions are given in the Methods section).

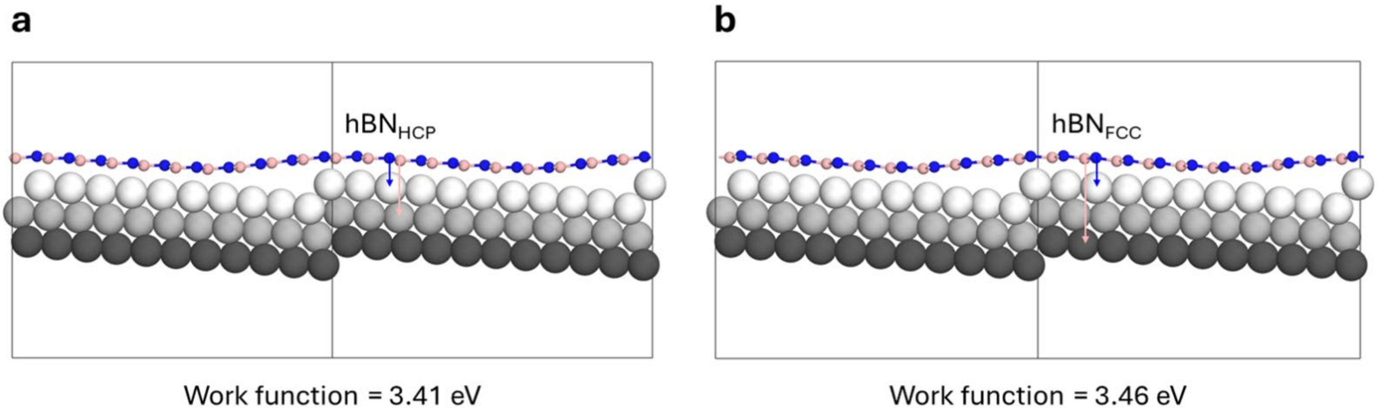

Fig. S6. Optimized structures of epitaxial hBN on Ni(5 5 6) surface, giving a work function difference of 0.05 eV.

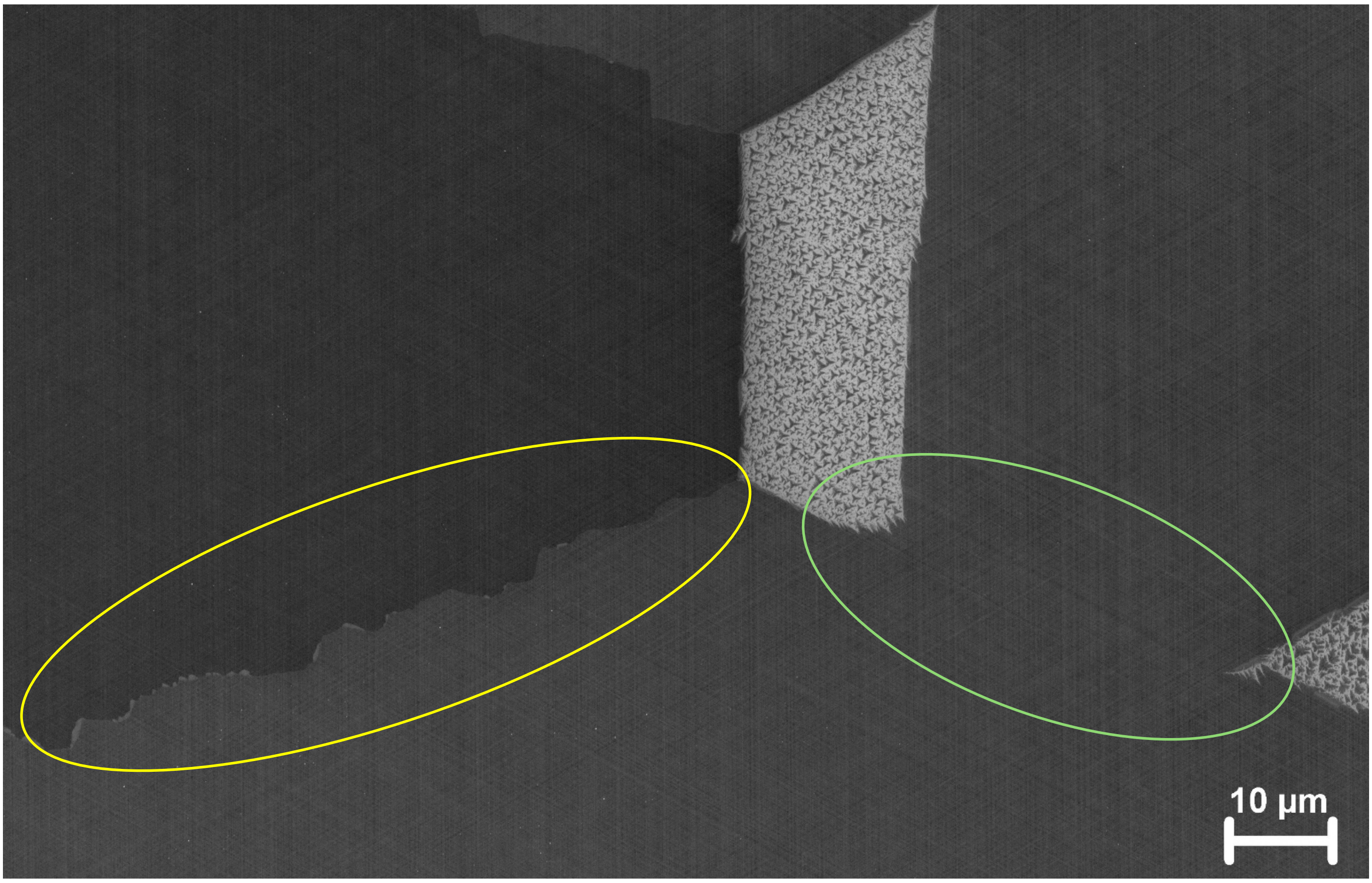

Fig. S7. Seamless stitching between two epi hBN domains of the same type (green ellipse) and bilayer growth (light colour) between two epi domains of different types (yellow ellipse). A magnified image of overlapping domain bilayer growth is shown in Fig. 3a. The CVD process conditions are given in the Methods section.

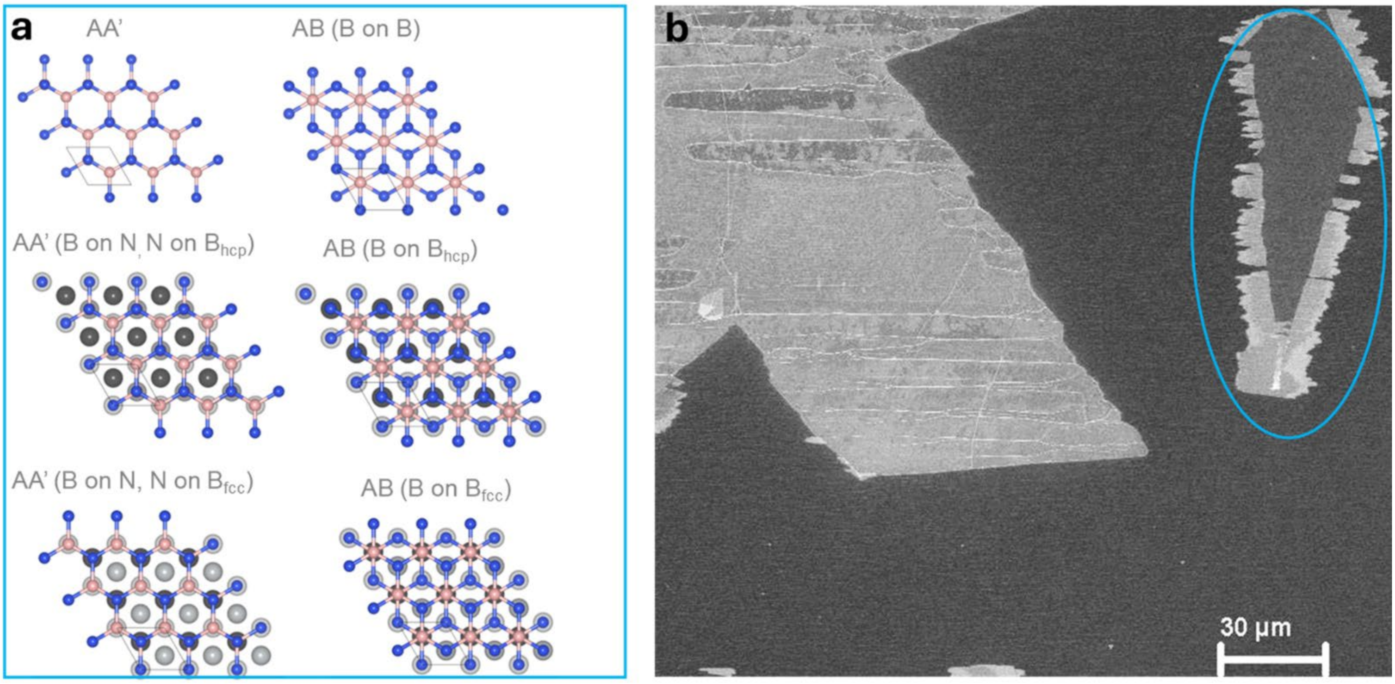

Fig. S8. Overlapping bilayers. (a) Other structures used in our DFT calculations, corresponding to Fig. 5a-c in main text. (b) hBN growth observations between the two epi hBN monolayers (dark grey and light grey) showing the bilayer region grows into the darker region ("HCP" configuration). The large lighter region on the left is the large area bilayer region that formed under twisted monolayer hBN upon prolonged borazine exposure, where the epi hBN monolayer grew under the twisted hBN monolayer. The CVD process conditions are given in the Methods section.

Model	Stacking	Total energy (eV)	Work function (eV)
free-standing hBN-hBN	AA'	-740.063	5.72
free-standing hBN-hBN	AB	-740.056	5.63
Ni(111)-hBN$_{HCP}$-hBN	AA'	-4867.738	3.26
Ni(111)-hBN$_{HCP}$-hBN	AB	-4867.733	3.13
Ni(111)-hBN$_{FCC}$-hBN	AA'	-4867.749	3.33
Ni(111)-hBN$_{FCC}$-hBN	AB	-4867.745	3.17

Table 2. DFT total energies and work functions of optimized local minima structures for free-standing bilayer hBN, Ni(111)-hBN$_{HCP}$-hBN and Ni(111)-hBN$_{FCC}$-hBN.

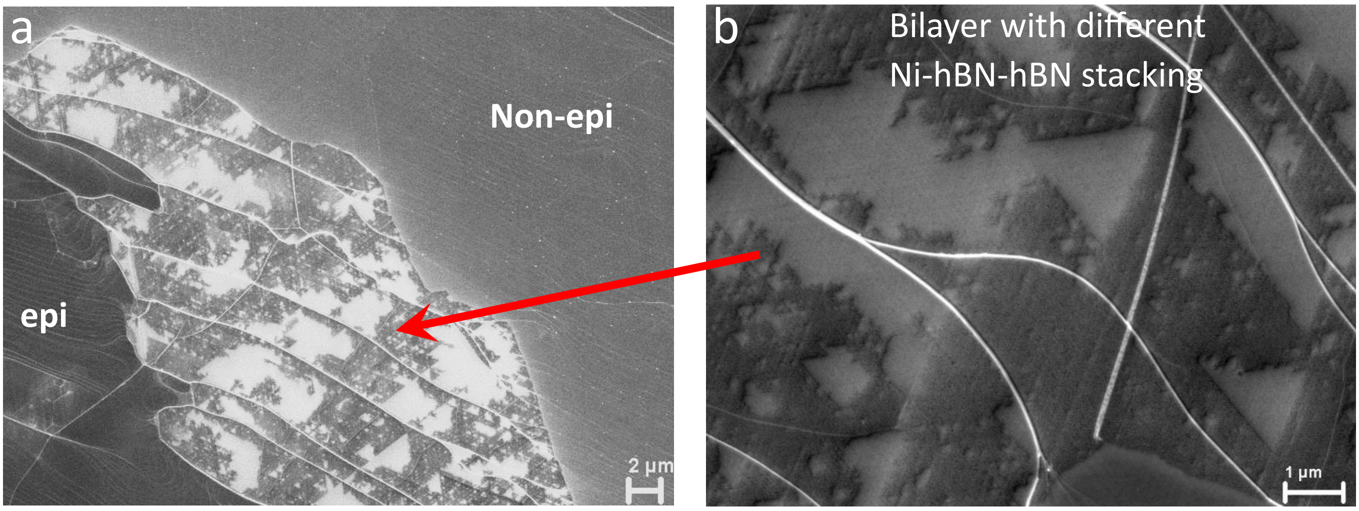

Fig. S9. Magnified overlapping bilayer between epi and non epi hBN regions showing triangular features, which we potentially attribute to different stacking between Ni and bilayer hBN. The CVD process conditions are given in the Methods section.

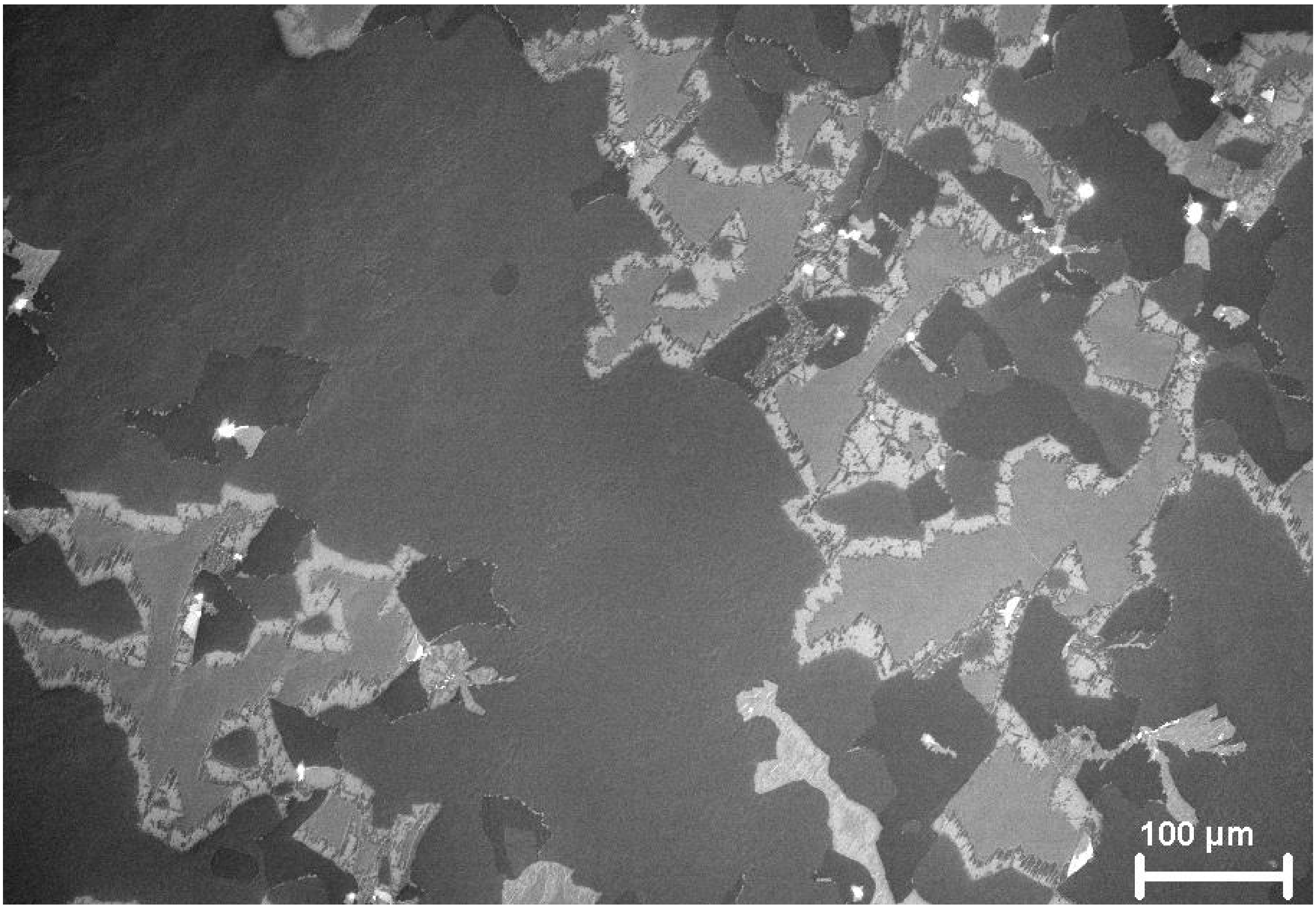

Fig. S10. Another example of a full coverage hBN film on Ni(111) consisting of different regions as discussed in main text. The CVD process conditions are given in the Methods section.

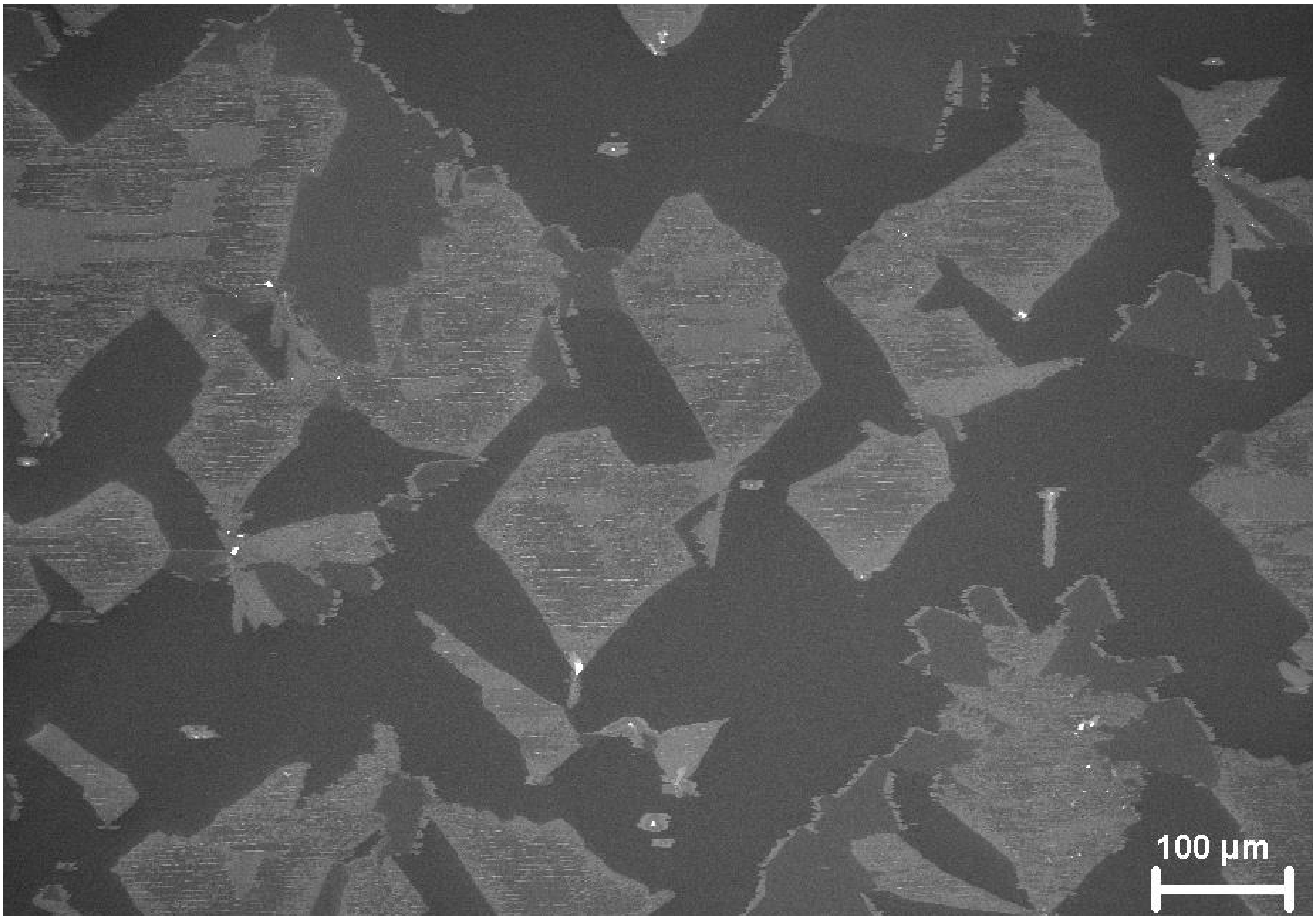

Fig. S11. Another example of a full coverage hBN film on Ni(111) consisting of different regions as discussed in main text. The CVD process conditions are given in the Methods section.

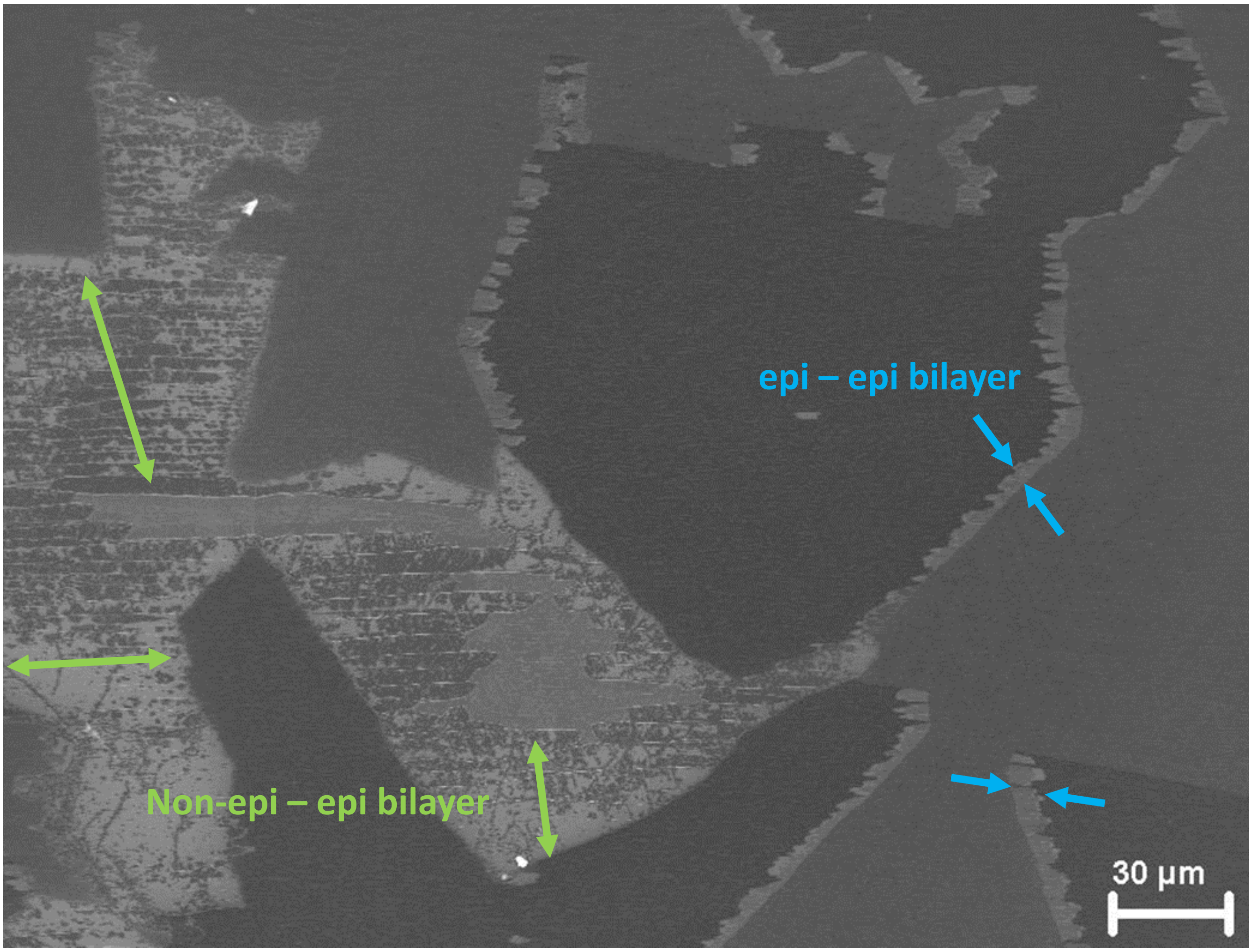

Fig. S12. Another example of a full coverage hBN film on Ni(111) consisting of different regions as discussed in main text. The difference in the growth rates of non-epi – epi bilayers, and epi-epi bilayers are shown with differently coloured arrows. The CVD process conditions are given in the Methods section.

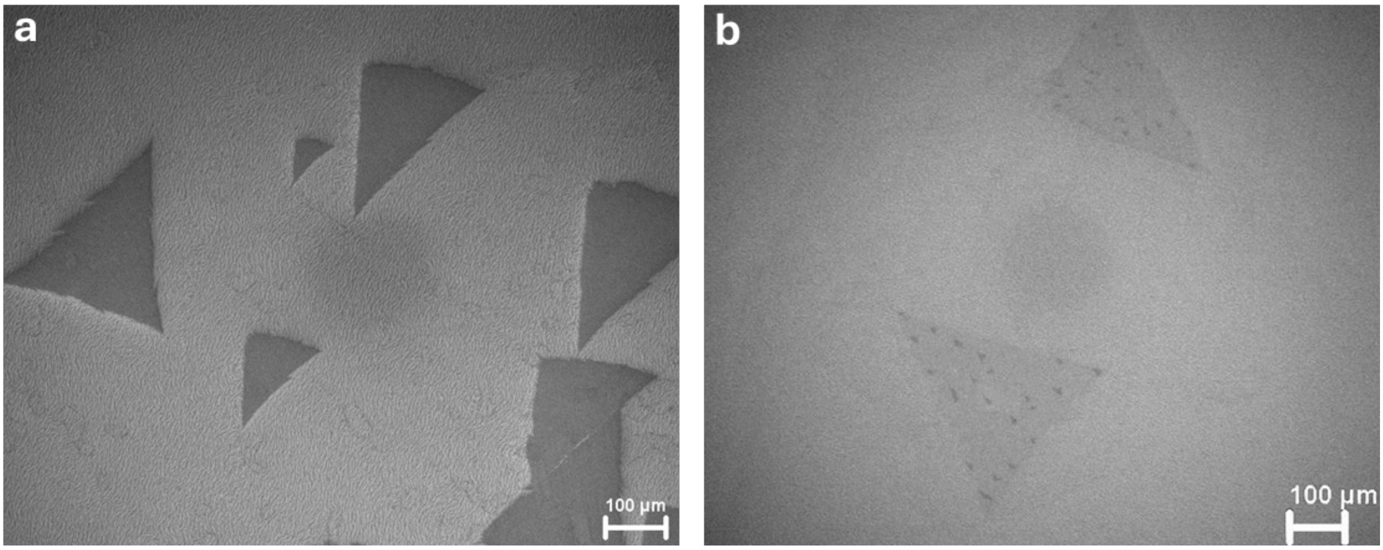

Fig. S13. Change in SE contrast after prolonged storage in air. (a-b) The same hBN/Ni(111) sample immediately after growth and after storage for 2 weeks in British air. The CVD process conditions are given in the Methods section.

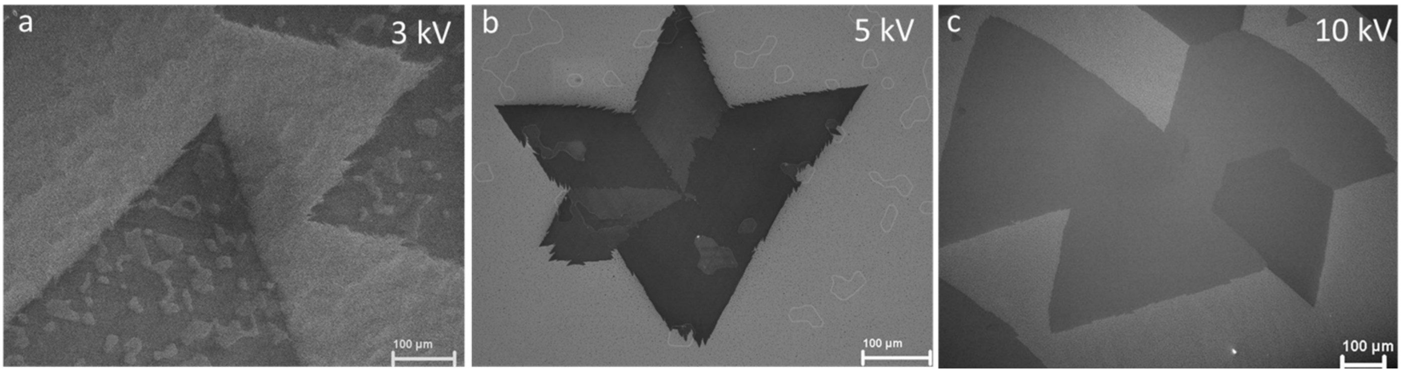

Fig. S14. hBN contrast between rotated hBN-Ni(111) either because of twinned Ni grains (small round inclusions) with a single hBN domain in (a) and (b), or contrast due to rotated hBN domains (large triangles) on a single crystal Ni film, in (b) and (c), under different accelerating voltages. Imaging conditions: (a) 3 kV, ~0.7 nA, 17.5 mm WD; (b) 5kV, ~0.7nA, 9.8mm WD; (c) 10 kV, ~0.7 nA, 10.4mm WD.

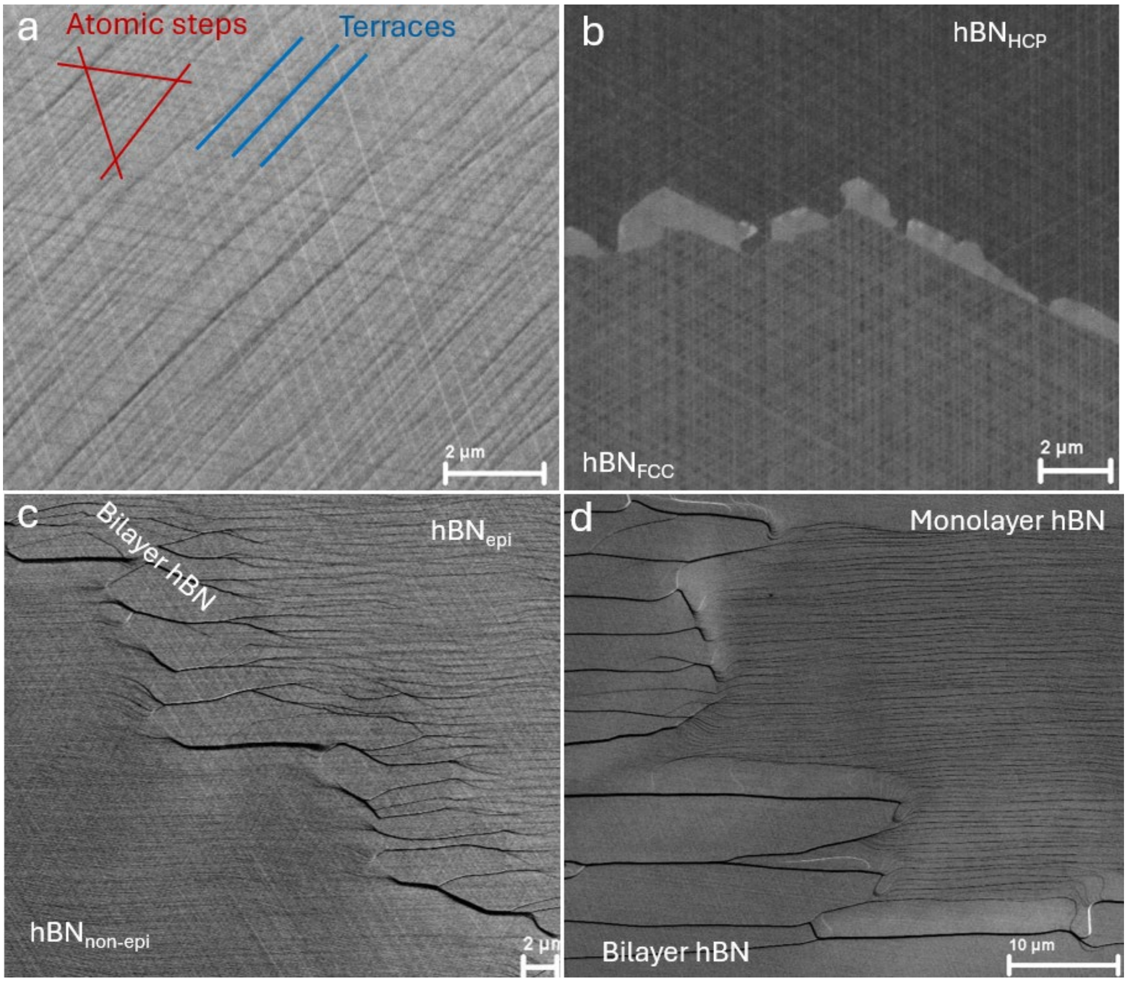

Fig. S15. Topography of hBN domains on Ni (111). (a) An image from the ET detector highlights the topography, that can be correlated with AFM in Fig. 3b. Epitaxial domains with triangular distributed atomic steps are observed. Such atomic steps are also observed on bare Ni (Fig. 3a). Furthermore, step-bunching into terraces is observed in one direction. (b) hBN epi domains (HCP & FCC) show similar topography. (c) Topography difference between epi and non-epi hBN domains. The non-epi domains appear smoother without obvious terraces. (d) Bilayer hBN domains show significantly larger terraces compared to monolayer domains. Imaging conditions: 5kV, ~0.25 nA, 10mm WD.

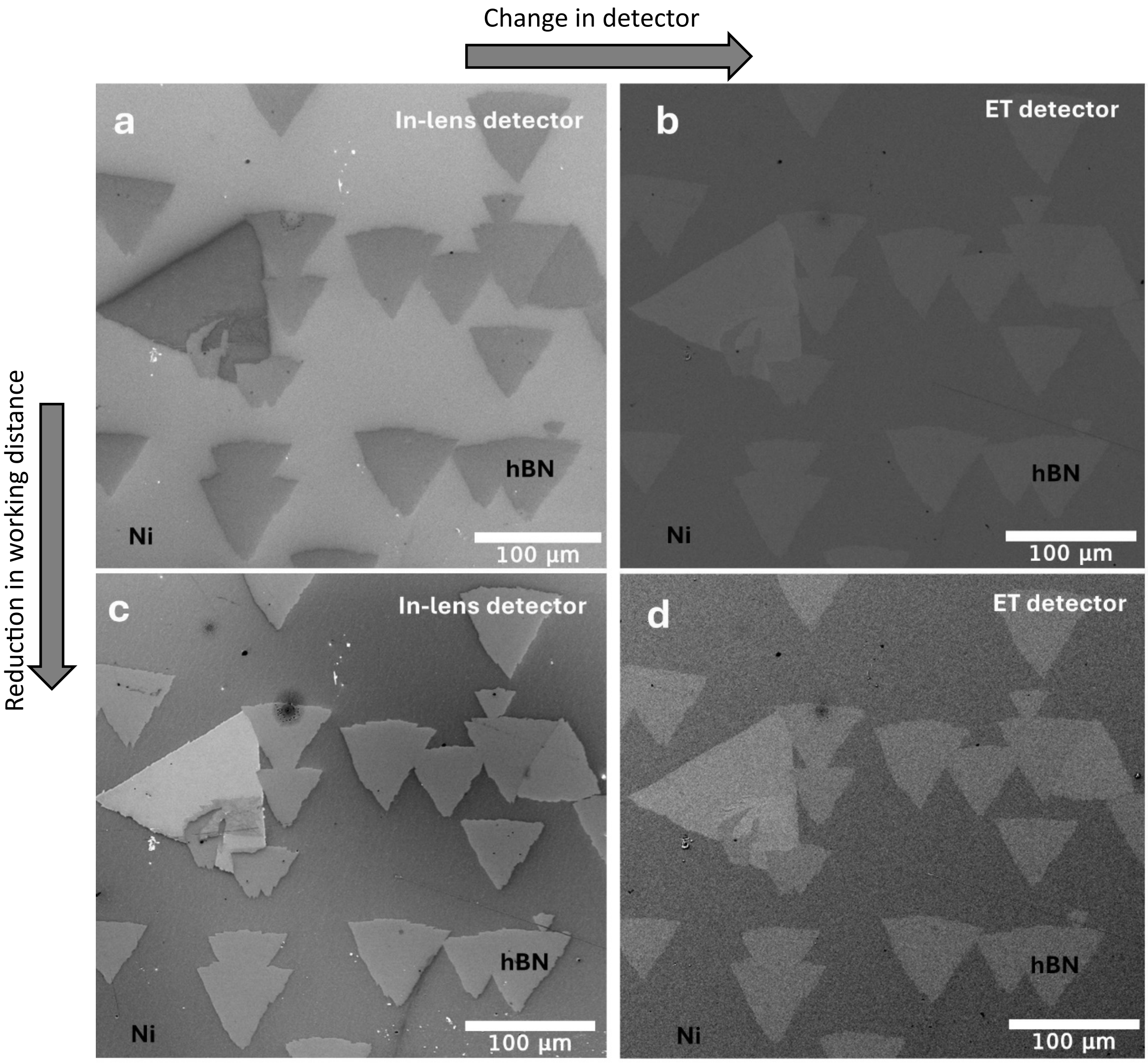

Fig. S16. Change in SE contrast after prolonged storage in air (> 6 month). Figures a and b were acquired at a working distance (WD) of 12.0 mm, while Figures c and d were captured at a shorter WD of 3.8 mm. Figures a and c utilise the in-lens detector, whereas Figures b and d were acquired using the ET detector. Accelerating voltage: 5kV, Aperture: 60 µm. We note that the level of Ni surface/interface oxidation will differ underneath the differently coupled hBN domains and for the "bare" Ni. This will affect the relative SE contrast.

References.

1. Babenko V, Fan Y, Veigang-Radulescu VP, Brennan B, Pollard AJ, Burton O, et al. Oxidising and carburising catalyst conditioning for the controlled growth and transfer of large crystal monolayer hexagonal boron nitride. 2d Mater 2020, 7(2)
